# Validation of eDNA methods for managing the terrestrial invasive snake *Lampropeltis californiae* on the Canary Islands

**DOI:** 10.1038/s41598-025-96387-8

**Published:** 2025-04-23

**Authors:** Mercedes López-González, Julien C. Piquet, Borja Maestresalas, Marta López-Darias

**Affiliations:** Spanish National Research Council (CSIC), Av. Astrofísico Fco. Sánchez, 3, San Cristóbal de La Laguna, 38206 Tenerife, Canary Islands Spain

**Keywords:** Canary Islands, Early detection, eDNA, eDNA degradation, Invasive snake management, *Lampropeltis californiae*, Invasive species, Conservation biology

## Abstract

Invasive snakes are among the most challenging invaders worldwide due to their exceptionally low detection rate and grave ecological impacts. Environmental DNA (eDNA) has emerged as a promising tool to improve invasive snake detection and enhancing management programs, yet its application to terrestrial snakes remains underexplored. This study provides the first advances in the use of eDNA techniques to detect the terrestrial invasive California kingsnake (*Lampropeltis californiae*). We designed *L. californiae*-specific primers and tested their effectiveness in detecting the species in different environmental samples, including swab samples from underneath artificial cover objects (ACOs) made of different materials, soil beneath ACOs, randomly collected soil, and researchers’ boots. Additionally, we conducted a controlled experiment to assess the accumulation and degradation rate of *L. californiae* eDNA over a 14-day period (7 with snakes in the terraria and 7 after having removed them). We detected *L. californiae* eDNA in 9.31% of swab samples, in 2.22% of soil samples under ACOs, and in 2.56% of boot samples, while no detections appeared in randomly collected soil or controls. In the controlled experiment, eDNA was undetectable in terraria prior snake introductions, but remained detectable throughout the study, with no evidence of snake eDNA degradation after snake removal. These findings provide key insights for the implementation of an eDNA-based protocol for the detection of *L. californiae* in Gran Canaria, offering a valuable tool for monitoring this invasive species. Furthermore, this study could be used for refining eDNA methodologies to detect other elusive terrestrial snake species elsewhere.

**Supplementary Information:**

The online version contains supplementary material available at 10.1038/s41598-025-96387-8.

## Introduction

Reliable information on the presence and distribution of invasive species is crucial for the planning, prioritization and implementation of effective conservation actions at different invasion stages^1,2^, ultimately leading to cost-effective interdiction and eradication efforts^3–5^. Long-term management programs targeting established invaders also rely on precise knowledge of species presence and distribution, as it contributes to the assessment of management feasibility, the planning and prioritization of conservation actions, the evaluation of program development and the confirmation of invasive species removal^5–7^. However, ascertaining species presence and distribution can be time and resource-consuming^7^, and failing to do so can have dire consequences for conservation programs^8–11^. The utilisation of techniques for the detection of environmental DNA (eDNA) in natural environments offers a promising solution to these challenges. This approach overcomes the limitations associated with traditional sampling methods^12,13^ and facilitates the identification of species irrespective of their life stage or abundance^14–16^. However, the implementation of eDNA protocols is contingent, among others, on the development of specific primers for the target species, the adaptation of sampling protocols to its ecological characteristics, and the evaluation of the species DNA degradation in the environment^12,13,17^. These fundamental requirements ensure the specificity and sensitivity of eDNA techniques for the target species, thereby preventing management from being misled by faulty detection protocols^12^. Despite the substantial progress that has been made in this regard for aquatic species^18^, the application of eDNA for terrestrial organisms is still in its early stages^19^.

Invasive snakes are among the most challenging biological invaders, posing a serious threat to global conservation goals. The increasing number of snakes transported through unintentional and deliberate pathways^20–22^ has led to the numerous established populations worldwide^23^ that cause irreversible ecological and socioeconomic damages^24^. Consequently, conservation practitioners must develop effective strategies to address this challenge, though the elusiveness of terrestrial snakes poses significant obstacles to their effective surveillance and management^8,25,26^. The use of environmental DNA is a promising solution to this problem, yet its application in the field of terrestrial snake research also remains to be investigated^27–30^.

We initiated the implementation of eDNA techniques in the management of the terrestrial California kingsnake *Lampropeltis californiae*. This species was introduced to the island of Gran Canaria (Canary Islands, Spain) more than two decades ago^31^, and has notably expanded its range since then, outpacing management efforts to control its spread^32,33^. The species management has been largely hindered by the extremely low probability of detection and capture rate of *L. californiae*, as it is a fossorial species that spends most of its time concealed underground and exhibits ephemeral and low activity patterns on the surface^34^. The primary conservation objective in the region is to control the species’ expansion and prevent its spread to other islands, as *L. californiae* is causing severe ecological impacts^35,36^ that are transferable to other islands, given the archipelago’s high suitability for the species^37^. In addition, the high probability of the species being introduced in continental Europe has led to its inclusion in the List of Invasive Alien Species of European Concern (Regulation (EU) 2022/1203), which makes it also a conservation priority at the European level. In this context, the primary objectives of this study were to (1) design and validate specific primers for *L. californiae*, (2) evaluate various sampling methods for collecting and detecting *L. californiae* eDNA in the field, and (3) analyze *L. californiae* eDNA accumulation and degradation to inform detection protocols. The sampling and laboratory protocols developed could be useful and informative for the implementation of eDNA techniques in the detection of other terrestrial snakes elsewhere.

## Methods

### Design and validation of *Lampropeltis californiae* specific primers

We designed primers for the cytochrome *c* oxidase I subunit (COI ≈ 654 bp) of *L. californiae* to distinguish this species from the co-existing reptiles of Gran Canaria, specifically the Gran Canaria giant lizard *Gallotia stehlini*, the Gran Canaria skink *Chalcides sexlineatus*, and Boettger’s wall gecko *Tarentola boettgeri*. *Lampropeltis californiae* and *T. boettgeri* are listed as least-concern species at the UICN Red List, while *G. stehlini* is classified as critically endangered and *C. sexlineatus* as endangered^38–41^. We extracted these species DNA from small tail fragments (approximately 30 mg) of four *L. californiae* individuals originating from the four primary invaded areas of Gran Canaria^33^, as well as two individuals of each endemic reptile species (own samples, collected on previous field campaigns), stored in 95% alcohol at − 20 °C, available in our facilities at the CSIC (Tenerife, Canary Islands). Following the manufacturers’ protocols, we extracted genomic DNA from *L. californiae* using the commercial kit E.Z.N.A.^®^ Tissue DNA (Omega Bio-tek Inc., USA), whereas we used ZR Genomic DNA Tissue MicroPrep Kit (Zymo Research, USA) to extract DNA from the endemic reptiles. We assessed DNA quantity and quality using a NanoDrop^®^ND-1000 spectrophotometer (Thermo Fisher Scientific, USA).

For COI amplification, we used the universal primers LCO1490/HCO2198^42^ for all species except for *T. boettgeri*, for which we employed RepCOIF/RepCOIR primers^43^. We performed PCR reactions in a Bio-Rad T-100 Thermal Cycler (BioRad, USA) in a 25 µL reaction volume containing 2.5 µL of T10x PCR TaKaRa (15 mM MgCl_2_) (Takara Bio Inc., Japan), 2 µL of dNTP mix (2.5 mM) (Takara Bio Inc., Japan), 1 µL (0.5 µg) of bovine serum albumin (BSA, BioLine, UK), 0.125 µL of TaKaRa Taq (1.25 U/µL) (Takara Bio Inc., Japan), 0.8 µL (10 mM) of each primer, and 20 ng of the extracted DNA. The PCR conditions for *L. californiae*, *G. stehlini* and *C. sexlineatus* consisted of an initial denaturation at 95 °C for 1 min, followed by 35 amplification cycles (15 s at 94 °C, 20 s at 50 °C, 30 s at 72 °C) and a final extension at 72 °C for 10 min. For *T. boettgeri*, we performed a touchdown PCR with slight modifications to the conditions established by Velo-Antón et al.^44^: an initial denaturation at 95 °C for 3 min, followed by an initial phase of 9 cycles (40 s at 95 °C, 30 s at 52 °C, decreasing by 0.5 °C per cycle to 48 °C, and 45 s at 72 °C), a second phase of 31 cycles (30 s at 95 °C, 30 s at 48 °C, and 45 s at 72 °C), and a final extension at 72 °C for 7 min. We visualized PCR products by electrophoresis on 1.7% agarose gel stained with Real-Safe (Real Biotech Corporation, Taiwan). We conducted the sequencing of fragments at the Genomic Service of the University of La Laguna. We aligned COI sequences (GenBank Accession numbers for species haplotypes: PQ870365, PQ867189, PQ867192, PQ867193, PQ870406) with available *Lampropeltis* sequences retrieved from GenBank (https://www.ncbi.nlm.nih.gov) and Bold Systems v3 (https://v3.boldsystems.org/) (see the Supplementary Information I) using ClustalW in MEGA X^45^. Given that shorter DNA sequences are more likely to persist in the environment and improve eDNA detection rates^46^, we used Primer-BLAST^47^ to design a short, species-specific sequence that distinguished *L. californiae* from endemic reptiles. We then tested primer specificity by amplifying previously extracted DNA from *L. californiae* and the endemic reptiles using the same PCR conditions as previously described, with an annealing temperature of 57 °C and the subsequent visualization of PCR products via 1.7% agarose gel electrophoresis stained with Real-Safe (Real Biotech Corporation, Taiwan).

Additionally, we amplified extracted DNA using quantitative PCR (qPCR) with 10–20 ng of DNA, 300 nM of each primer, and 7.5 µL of iTaq Universal SYBR Green Supermix (Bio-Rad, USA) in a 15 µL reaction volume. We performed reactions on an iCycler BIO-RAD real-time thermal cycler at the Genomic Service of the University of La Laguna. In order to optimize amplification conditions, we first tested a gradient of temperatures and cycle numbers using *L. californiae* DNA as the positive control and a sample without DNA as the negative control. The final qPCR conditions comprised an initial denaturation at 95 °C for 10 min, followed by 40 amplification cycles (15 s at 95 °C, 20 s at 57 °C, and 30 s at 72 °C). We assessed primer specificity by comparing qPCR melting curves, ensuring that *L. californiae*-specific primers produced distinct melting curves, while non-specific amplification resulted in spurious curves for endemic reptiles.

### Detection of *Lampropeltis californiae* eDNA in environmental samples

**Fig. 1:**
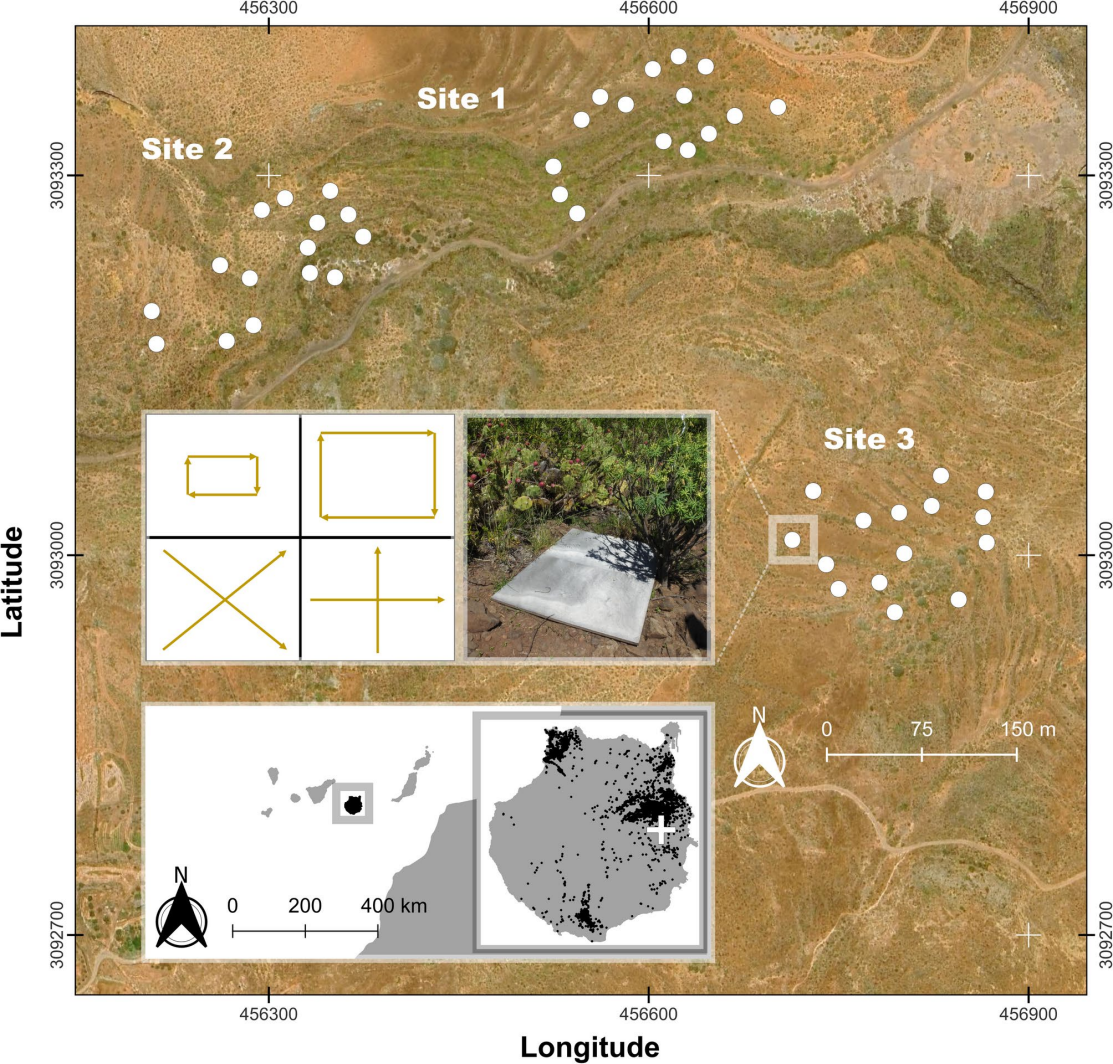
Map of the study area showing the location of our sampling sites and artificial cover objects (ACOs). We visited ACOs to collect swab samples following the patterns described in the top inset (adapted from Matthias et al.^30^), and soil samples from the substrate directly under their corners and center. We also collected soil samples from random locations within each site > 20 m apart from each other and each ACO. The second inset shows the location of the Canary Islands and the island of Gran Canaria within the archipelago, as well the location of the study area (write cross) and *Lampropeltis californiae* accumulated captures and sightings from 2009 to 2024 according to the control program^33^ (black dots).

We conducted an eDNA detection field experiment in the surroundings of Cuevas de Calacio (Telde), an area occupied by *L. californiae* since 2017^33^, primarily covered by native xeric shrublands^48^ and experiencing mild weather conditions^49^. We used QGIS 3.20^50^ to delineate three sampling sites of similar size (1.4 ha) where the highest concentration of *L. californiae* catches had occurred during the previous years, according to control program database^33^. These sites were located at least 120 m apart to account for the home range of *L. californiae*^34^ (Fig. 1). At each site, we established 15 random locations using the QGIS 3.34 random point generator^50^, separated a minimum of 20 m to avoid spatial overlap and based on the most frequently daily movements of *L. californiae*^34^. From May 4th to 5th 2022, we placed three of each of these artificial cover objects (ACOs) types in the generated random locations: plywood boards (1.22 × 0.80 m), corrugated bitumen sheets (1.20 × 0.70 m), corrugated steel plates (1.20 × 0.70 m), flat bitumen sheets with a reflective top side (1.00 × 1.00 m), and rubber sheets (1.00 × 1.00 m). To identify the most effective sampling approach to detect snake eDNA, we compared two sampling regimes. We executed the first one from January 30th to June 8th 2023, encompassing nine one-day sampling sessions, 30 days apart in January and February, and 15 days apart subsequently. During this period, we collected a swab sample from the underside of one randomly selected ACO from each type at each site, as well as from the soil immediately beneath the selected ACOs. Additionally, we collected soil samples at random locations that we placed prior to each sampling session using QGIS 3.20 random point generator^50^ and located at least 20 m away from each sampling point. During the initial period, we also collected swab samples from the soles of researchers’ boots at the end of each session to assess the viability of this technique as an alternative detection method. Despite the fact boots might not provide accurate location of eDNA presence, it could be used to detect the species in given areas by simply walking through them. We performed the second sampling regime from June 8th to June 30th, consisting of four one-day sampling sessions, seven days apart, during which we collected swab samples from all ACOs at each site.

We collected ACO swab samples following a protocol adapted from Matthias et al.^30^ and Kyle et al.^19^). We used sterile cotton discs previously moistened with 70% isopropanol, which we gently rubbed^30^ along a small square midway between the edge of the ACO and the center, starting at the top left, then making an “X” pattern connecting each corner, followed by a “+” pattern, and finishing with a square along the outer edges of each ACO^30^ (Fig. 1). We collected boot samples by gently rubbing a cotton disc over the entire surface of each team member boots’ soles at the end of each sampling session, with each boot sample being stored separately. Each soil sample consisted of a 50 mL Falcon tube filled with small amounts of soil collected by sliding the tube directly under the four corners and the center of the soil covered by each ACO and over an area equivalent to that of the ACO for random soil samples. At the end of each sampling session, we moistened a cotton pad with 70% isopropanol and stored these as negative field controls. We transported all samples back to the laboratory on the same day of sampling and stored them at − 20 °C in a freezer drawer that had not previously contained samples of the target species. In an effort to minimize contamination during the entire field sampling workflow^51^, we used new disposable latex gloves to collect each sample, stored all swab samples in individual paper envelopes, and separated each sample into small, individual, hermetic sealed plastic bags. We incorporated silica gel beads into the swab and boot bags with the objective of mitigating potential mold growth. Subsequent to the collection of samples, we consolidated the small individual plastic bags into larger hermetic bags for each sample type and site. No contact with the target species had occurred prior to or during the sampling day, since all sampling and personal material (in addition to the researchers) travelled to Gran Canaria from the uninvaded island of Tenerife for each sampling session.

We extracted DNA in a pre-PCR laboratory located in the CSIC facilities in Tenerife (no samples of the target species had been recently processed there) and following a specific protocol for each sample type. For swab samples, we separated the most superficial layer of the cotton disc using sterilized forceps and scalpels for each sample and placed it into a 1.5 mL tube. We conducted the procedure for soil samples in accordance with the protocol proposed by Matthias et al.^30^. We shook the samples and transferred 15 mL of their content to another 50 mL Falcon tube, which we then filled with ultrapure water (Ultra-Pure Water System, Milli-Q^®^, Merck, USA) up to 45 ml. We then inverted and shook vigorously the Falcon tube for *c.* 30 s. We left the tube in a vertical position at 4 °C for 24 h to allow the sample to settle. We transferred the supernatant to a sterile single-use 250 mL vacuum filtering funnel (Pall Life Science, USA), and used sterile cotton gauzes to separate larger stones and plant debris. We filtered the supernatant on a filtration ramp (Millipore^®^, USA) with 47 mm polystyrene membrane filters and 0.45 μm pores (Prat Dumas, France). We then extracted the filter from the ramp, cut a quarter of it, and placed this final sample in a 1.5 mL tube. We extracted the DNA retained in the cotton discs and in the filters using the commercial kit E.Z.N.A.^®^ Tissue DNA (Omega, Bio-tek Inc., USA), following the manufacturer’s protocol. In this process, we ensured that the entire inner surface of the discs and filters were in contact with the lysis buffer. To amplify *L. californiae* eDNA, we employed the qPCR (utilizing the same conditions as previously described), due to its higher sensitivity to small-sized DNA fragments in comparison with conventional PCR, making it a more suitable for eDNA^16,52,53^. This technique is frequently employed for the detection of single species^13,54^, offering enhanced detection probabilities in comparison to metabarcoding for species-specific assays^55,56^. In accordance with the approach outlined by Dejean et al.^57^, we amplified three replicates of each sample, with the inclusion of positive and negative controls. We designated a sample as positive if (1) at least one of the replicates generated a melt curve within ± 0.5 °C of that of *L. californiae*, which we identified during the primer selection phase, and (2) the cycle threshold value (Ct) of 40, set as the detection limit, coincided with that of *L. californiae*^28,58^. To reduce subjectivity in the interpretation of melt curves, two researchers evaluated the results, and only samples with clearly similar melting curves and Ct values to those of *L. californiae* were considered as positive (we repeated those that were ambiguous).

To minimize contamination during laboratory procedures, we handled DNA isolates and prepared qPCR reactions using filtered pipet tips and under the laminar flow hood in the pre-PCR laboratory. We performed all tasks in a dedicated working area within the pre-PCR laboratory, which was thoroughly cleaned with 10% diluted bleach after processing each sample. We used new disposable gloves to manipulate each sample and to access to the freezer, and all instruments were sterilized with 10% diluted bleach, rinsed with distilled water and dried with disposable paper towels. We carried out amplifications in a post-PCR laboratory within the CSIC facilities that did not share equipment or material with the pre-PCR laboratory. We transferred samples between pre- and post-PCR laboratories in a single direction (pre-PCR to post-PCR), and we spaced the pre-PCR and post-PRC work at least 24 h apart.

Finally, we compared detection rates obtained only from ACO swab samples among periods, sites and types of materials using Chi square tests. We used the package *chisq.posthoc.test*^59^ to retrieve standardized residuals, and obtain cell-specific *p-*values as a *post hoc* test following Beasley and Schumacher^60^. We performed all data analysis using R 4.4.1^61^.

### Accumulation and degradation of *Lampropeltis californiae* eDNA

To analyze the accumulation and degradation of *L. californiae* eDNA, we designed a pilot experiment under controlled conditions following Kucherenko et al.^27^. The experiment set-up and sample collection were performed by the staff of the public company responsible for the control program, GESPLAN S.A.. On May 7th 2023, GESPLAN S.A. personnel undertook a thorough cleaning of four glass terraria (48–78 cm × 38–60 cm × 49 cm; length × width × height) using a commercial disinfectant soap and 10% diluted bleach, after which the terraria were filled with 2 L of a commercial substrate for reptiles (Repti-Selva Sand, ICA S.A., Spain). On the subsequent day, GESPLAN S.A. staff collected a sample of substrate (negative control) filling up a 2 mL tube with a similar amount of substrate from each corner and center of each terrarium. At 7 a.m. of the same day, they placed one adult individual *L. californiae* in each terrarium, collecting a substrate sample every hour onwards until 5 p.m. of the same day and one sample per day in the early morning until day 7, when they removed the snakes. They continued to collect substrate samples once a day from day 8 to day 12, with a final collection on day 14, as performed by Kucherenko et al.^27^. All substrate sample collection were carried out with care to prevent direct contact with visible urine or feces. Potential sample contamination was minimized by using new disposable latex gloves for each sample and storing them in a − 20 °C freezer located outside the facilities where terraria were situated and where no samples of *L. californiae* had been previously stored. We transported all samples to the CSIC laboratory facilities for subsequent analysis.

We extracted the DNA in the CSIC laboratories using a self-designed protocol with the following steps: firstly, we transferred the contents of each sample tube into a new 2 mL tube, which we then stored at − 20 °C; secondly, we rubbed a sterile cotton swab moistened with ultrapure water (Ultra Pure Water System, Milli-Q^®^, Millipore^®^, USA) over the walls of the empty tube; thirdly, we cut the tip of the swab and dropped it into a 1.5 mL tube. This protocol reduced the time of sample processing without compromising the sensitivity of the technique (tested against other two alternative sample processing methods; see results of those other methods in Supplementary Information II). We extracted DNA from the swab tips using the commercial extraction kit E.Z.N.A.^®^ Tissue DNA (Omega, Bio-tek Inc., USA), following the manufacturer’s protocol.

We utilized conventional PCR for the amplification of extracted DNA from samples collected during the initial phase in the terraria (days 1 to 7), whereas we employed qPCR for subsequent samples collected after snake removal. We used these two approaches due to budgetary and logistical constraints, and used qPCR to better characterize the degradation of eDNA. We performed conventional PCRs in a Bio-Rad T-100 Thermal Cycler (BioRad, Hercules, USA) using a total volume of 20 µL containing 2 µL of T10x PCR TaKaRa (15 mM MgCl_2_) (Takara Bio Inc., Japan), 1.6 µL of dNTP mix (Takara Bio Inc., Japan) (2.5 mM each), 0.8 µL (0.5 µg) of BSA (BioLine, UK), 0.64 µL (10 mM) of each primer, 0.125 µL of TaKaRa Taq (1.25 U/µL) and 2 µL of the extracted DNA. The PCR conditions comprised and initial step of 1 min at 95 °C, followed by 35 amplification cycles (15 s at 94 °C, 20 s at 57 °C, and 30 s at 72 °C), and a final 10 min extension at 72 °C. We visualized the amplification results with 1.7% agarose gel electrophoresis with Real-Safe (Real Biotech Corporation, Taiwan). We replicated previously specified qPCR conditions to amplify three qPCR replicates for each sample, in addition to positive and negative controls. We undertook the evaluation of DNA degradation by exploring Ct values over time, a semi-quantitative value that is inversely related to the amount of DNA in the sample^62,63^.

### Ethical statement

All procedures were approved by the Government of the Canary Islands under permit no. AGPA/11031/2023.

## Results

### Design and validation of *Lampropeltis californiae* specific primers

**Fig. 2:**
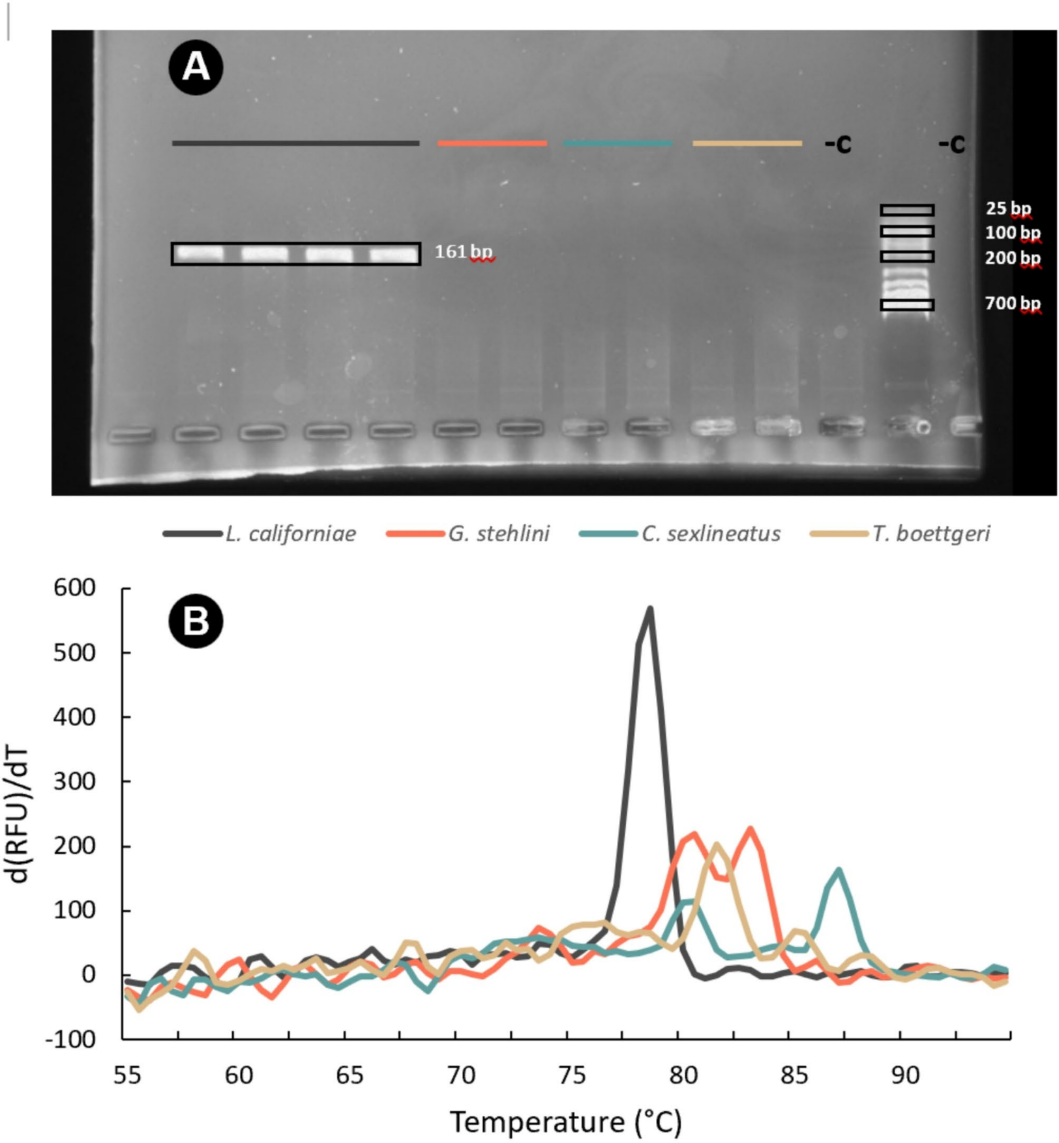
Specificity of the self-designed primers for the cytochrome oxidase I subunit. Panel **A** shows the amplification products (≈ 161 bp) obtained from the extracted DNA of *Lampropeltis californiae*, *Gallotia stehlini*, *Chalcides sexlineatus* and *Tarentola boettgeri* and visualized through 1.7% agarose gel electrophoresis with Real-Safe (Real Biotech Corporation, Taiwan) (the third-last and last lanes represent the negative controls (-C) and the second-last lane included the 25–700 bp DNA marker (LADD-DN1-500; OXGEN™). Panel **B** shows the melting temperature curves obtained through qPCR when amplifying the extracted DNA from tails of the four species.

The designed primer pair (LampCOIF: 5′-TTGGGGCCTGCCTAAGTATC-3′ and LampCOIR: 5′-GGGGAAGGCTATATCGGGAG-3′) enabled the differentiation of *L. californiae* from other co-occurring reptiles. We confirmed the specificity of the assay through the detection of target amplicons (161 bp between primers) in agarose gel electrophoresis for *L. californiae* (Fig. 2A), as well as through the clear differentiation of melting curves for *L. californiae* and endemic reptiles (Fig. 2B). We did not observe primer dimers, reagent inhibition (all positive controls amplified), nor contamination (no negative controls amplified) during the amplifications.

### Detection of *Lampropeltis californiae* in environmental DNA samples

We collected a total of 290 ACO swab samples (134 during the first sampling period and 170 during the second period, with 14 samples shared between both), as well as 135 soil samples underneath the ACOs, 135 soil samples in random locations, and 39 boots’ samples (see Table 1 for details). Despite observing one active snake and several fresh skin sheds during the sampling sessions, we did not detect *L. californiae* individuals or physical traces under the ACOs throughout the entire sampling period. During the initial sampling period, we detected *L. californiae* eDNA in 8.96% of swab samples, 2.22% of soil samples collected underneath the ACOs, and 2.56% of boot samples. However, the species remained undetected in randomly collected soil and negative controls. Of the 16 samples that were positive for *L. californiae* during this period, 18.75% were positive in one qPCR replicate, 50.00% in two replicates, and 31.25% in all three replicates. We only detected eDNA in swabs and soil under the same ACO on the same day in one instance. Of the 12 snake detections in swab samples, 41.67% occurred in corrugated bitumen sheets, 25.00% in corrugated steel plates, 25.00% in plywood boards, and 8.33% in rubber sheets (Table 1). Sampling site 2 accounted for 75.00% of all detections, with sampling sites 1 and 3 producing 8.33% and 16.67% of all snake detections, respectively. During the second sampling period, we detected *L. californiae* in 9.41% of all swab samples, 25.00% of which had one positive qPCR replicate, 12.50% had two positive replicates and 62.50% had all three positive replicates. In this period, 62.50% of all detections occurred in corrugated steel plates, 25.00% in corrugated bitumen sheets, and 12.50% in plywood boards. The spatial distribution of snake detections during the second period exhibited a similarity to that of the first period, with 50.00% of all detections occurring in sampling site 2, 37.50% in sampling site 1, and 12.50% in sampling site 3.

**Table 1:** Total number (Positives) and percentage (%) of samples in which we detected *Lampropeltis californiae* eDNA, and total number of samples analyzed (N). Samples include swab samples collected from artificial cover objects (ACOs) from January 30th to June 8th 2023 (ACOs 1; first sampling period) and June 8th to June 30th 2023 (ACOs 2; second sampling period) (see main text for further details), soil samples collected directly underneath ACOs (Soil ACOs) and at random locations within sampling sites (Soil random), and swab samples from the sole of team members’ boots (Boots). We also show the number of positive detections per type of ACO (ST = corrugated steel plate, BS = bitumen sheet, RU = rubber sheet, CBS = corrugated bitumen sheet and PL = plywood board) and sampling sites (1–3). For the purpose of total ACO counts, we included June 8th once (this day was shared between the two sampling periods).

	General data			Material					Sites		
	Positives	%	*N*	ST	BS	RU	CBS	PL	1	2	3
ACOs 1	12	8.96	134	3	0	1	5	3	1	9	2
ACOs 2	16	9.41	170	10	0	0	4	2	6	8	2
Total ACOs	27	9.31	290	13	0	1	9	4	7	17	3
Soil ACOs	3	2.22	135	0	0	0	1	2	2	0	1
Soil random	0	0.00	135						0	0	0
Boots	1	2.56	39								

Over the course of the two distinct sampling periods, we detected the presence of *L. californiae* in 9.31% of swab samples (Table 1). A 48.15% of swab positive detections were on corrugated steel plates, 33.33% on corrugated bitumen sheets, 14.82% on plywood boards, and 3.70% on rubber sheets (no detection on flat bitumen sheets). The snake detections were linked to 12 specific ACOs, seven of which detected snakes 2–5 times over the whole sampling period. A total of 62.96% of all snake detections through swabs belonged to site 2, while sites 1 and 3 produced 25.93% and 11.11% of all detections, respectively (Table 1). We did not detect positive samples in January, whereas February and March exhibited the highest percentage of positive detections (20.00% and 13.33%, respectively) (detection rate ≤ 10.00% for the remaining months).

We did not find significant differences in eDNA detection rate between periods for swab samples ($\chi_{1}^{2}=$ 0.01, *P* = 1.000). However, we observed a significant variation in the detection rate among sites ($\chi_{2}^{2}=$ 13.07, *P* = 0.001) and types of materials ($\chi_{4}^{2}=$ 25.39, *P* < 0.001) for all periods combined. Site 2 had a substantially higher detection rate compared to the other sites (standardized residual of 3.46, *P* = 0.003), while site 3 demonstrated a significantly lower rate (standardized residual of -2.65, *P* = 0.048). Corrugated steel plates showed a higher detection rate compared to the remaining materials (standardized residual of 3.98, *P* < 0.001).

### Accumulation and degradation of *Lampropeltis californiae* eDNA

**Fig. 3:**
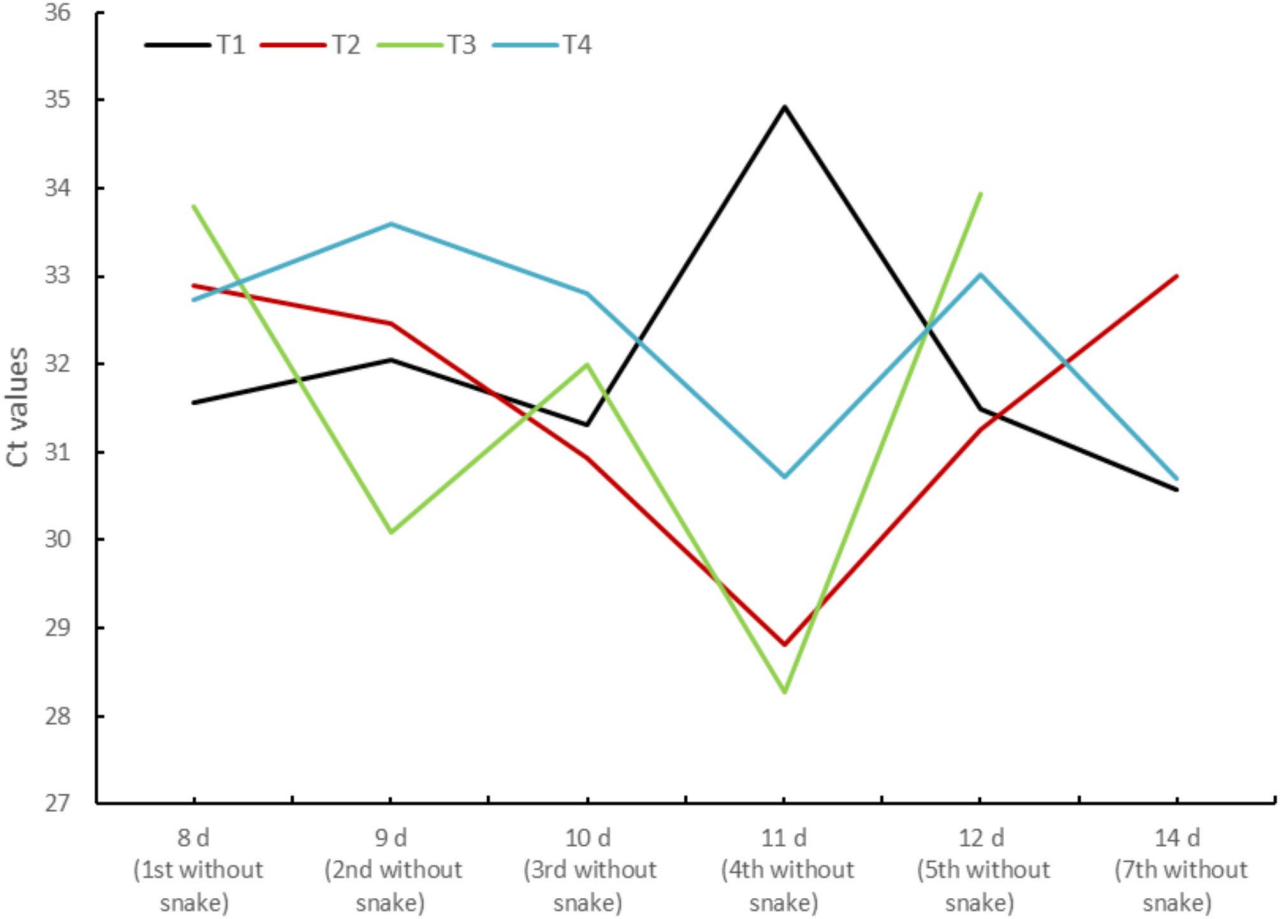
Cycle threshold (Ct) values per each terrarium (T1–T4) in the controlled condition experiment obtained through qPCR from day 8 to 12 and day 14 (following Kucherenko et al.^27^), and used to analyze eDNA degradation over time after *Lampropeltis californiae* individuals were removed from terraria.

The eDNA accumulation and degradation experiment yielded a total of 92 samples (23 per terraria). We did not detect *L. californiae* in any terrarium prior to release of snakes (Supplementary Information III). Positive samples appeared from the first hour onwards until the snakes were removed from the terraria, yet we obtained several negative samples on intermediate days in all terraria (Supplementary Information III). The use of qPCRs led to the detection of *L. californiae* in all samples and terraria, with the exception of a sample collected in one terrarium on day 14. We did not detected *L. californiae* eDNA in any of the negative controls (Supplementary Information III). Among positive samples, 4.35% had one positive replicate, 17.39% exhibited two positive replicates, and 78.26% were positive in all three replicates. We did not detect any evidence of eDNA degradation, as Ct values from the eighth day to the end of the experiment did not progressively increase over time in any terrarium (Fig. 3).

## Discussion

Our results suggest that the primers employed for the detection of *L. californiae* eDNA through the COI subunit exhibited specificity and can be utilized for conservation purposes in the Canary Islands. This is evidenced by the effective amplification of *L. californiae* DNA, as indicated by both gel electrophoresis and qPCR evaluation, and the lack of amplification for the endemic reptiles, as well as through the discrimination of *L. californiae* eDNA from all sympatric reptiles using the variation in melting temperature curves. Ensuring sensitivity and specificity is a crucial step in the effective application of eDNA techniques^14,64^, as false positives or false negatives may lead to misguided management outcomes^12^. Therefore, this represents a significant initial advance in the utilization of eDNA techniques for the management of *L. californiae* invasion. The focus of our detection protocol on the COI subunit was driven by its high interspecific variability^65^ and availability of a substantial reference database for this region^66^. However, environmental samples can contain eDNA sequences from numerous taxa at concentrations that may exceed those of the target species^13,64,67^. Therefore, even in the absence of any other snakes in Gran Canaria (with the exception of the Brahminy blindsnake *Virgotyphlops braminus*^68^, which is relegated mainly to gardens) it is advisable to further validate primer specificity before implementing widespread eDNA protocols. This is of particular importance if these primers are intended for use in other geographical areas, where mismatches with other untested species could occur^69^. Future tasks to improve qPCR specificity and sensitivity include designing a specific probe, in addition to an assay and dilution series of the target species^69^. The validation of primer specificity in the future would serve to substantiate the applicability of this technique, elucidate its strengths and limitations, and inform the conclusions that can be drawn from it^14,64,67^. In the event that the COI subunit generates primers that lack specificity when compared with a more extensive array of taxa, the use of alternative genetic markers should be investigated^70^.

The diverse sampling protocols employed in this study revealed that swabbing ACOs with cotton can be an effective method for detecting *L. californiae* eDNA, despite the absence of snakes under ACOs throughout the entire sampling period. This detection rate is comparable to that reported by Matthias et al.^30^ for the elusive and native sharp-tailed snake *Contia tenuis* (9.30%), who also employed swabs under ACOs and detected eDNA in locations where snakes had not been recently observed. The employment of ACOs constructed from diverse materials further enhanced the detection rate, achieving 15.48% and 11.49% with corrugated steel plates and corrugated bitumen sheets, respectively, the highest rate reported for terrestrial invasive snakes. This observation suggests that certain materials may exhibit superior efficacy in retaining eDNA, given the apparent lack of a clear preference for the use of specific cover materials in reptiles^71^. This result emphasizes the importance of selecting appropriate materials for DNA detection^19,72^. Consequently, future studies should focus on enhancing the detection rates of *L. californiae* (and other terrestrial snakes) in the field, which could be achieved by using more effective materials and studying the environmental conditions they can provide^19^. The detection rate obtained for soil samples collected under the ACOs (2.22%) was marginally higher than that reported for other species under similar conditions^28^, yet the low prevalence of snake eDNA in these samples compromises the usefulness of this sampling technique. The success of vertebrate eDNA detection is contingent on social organization, species behavior, and species biomass^73^. Given that *L. californiae* is a fossorial species that spends the majority of its time underground, travels short distances, has small home ranges^34^, and shows a strong preference for areas covered by vegetation and rocks (Maestresalas et al. *under review*), the location of ACOs within the home range of a particular snake or in favorable microhabitats could explain the clustering of most of the detections. Alternatively, the recurrence of positive detections in the same ACO may be attributable to a low rate of eDNA degradation, a finding that aligns with the outcomes of our controlled experiment and with the duration of up to two weeks after visiting an ACO of the eDNA from the little brown skink *Scincella lateralis*^19^. However, Kucherenko et al.^27^ did find eDNA degradation of red cornsnakes (*Pantherophis guttatus*) after six days of snake removal in a controlled laboratory setting similar to ours. Nevertheless, the lack of degradation in our controlled experiment, as well as the presence of negative samples on intermediate days of the experiment, may indicate potential errors in the sample processing that should be investigated further. Despite the meticulous efforts of the GESPLAN S.A. staff in avoiding contamination during sampling, the terraria were situated within the same facilities where all control program operations were conducted. This raises the possibility of other sources of *L. californiae* eDNA within these facilities. Therefore, future research should employ fine eDNA detection and quantification techniques to enhance our understanding of eDNA accumulation and degradation processes. While the present research offers encouraging initial progress in enhancing *L. californiae* detection using eDNA and unveiling the potential of this technique for the management of other elusive and terrestrial snakes elsewhere, there is still room for improvement and the development of more effective protocols. For instance, a higher detection rate may be achieved through the utilization of alternative environmental samples, such as vegetation^74^, rocks or invertebrate-borne DNA (iDNA)^75^. Given that the majority of Gran Canaria experiences semiarid conditions with limited water availability^49^ and all *Lampropeltis* species necessitate regular access to water or humid microhabitats^76^, the deployment of temporary water troughs in invaded areas, mimicking ponds used to detect *P. molurus bovittatus* in Florida^77^, could also provide favorable detection rates. This approach would facilitate the implementation of the well-established laboratory protocols designed for aquatic samples^45,78^. It would also be worthwhile to ascertain whether the probability of detection could be improved by extending the cotton disc in the ACO-based protocol or by increasing the quantity of soil collected or the number of sample replicates analyzed^29,30^. A more in-depth exploration of this technique could also allow for the investigation of the correlation between eDNA detection and species abundance or biomass^79,80^, as their link is still poorly understood for terrestrial environments^81^. In the present case, while we obtained a higher number of detections at the sites where GESPLAN S.A. personnel captured the highest number of individuals, this relation cannot be reliably analyzed due to the unavailability of information on the effort made at each site^33^. However, this result offers encouraging perspectives on the effectiveness of these methods to estimate density or abundance, critical parameters for invasive species management^2,5^.

The present research has the potential to enhance the capacity to detect *L. californiae*, thereby facilitating the determination and monitoring of the species’ distribution on the island. At present, this information can only be deduced from opportunistic detections^33,82^. The generation of reliable information would pave the way for the delineation and tracking of the invasion front^83^, and assist in the early detection of new populations^84^. The establishment of cost-effective detection protocols could also help in the implementation of much-needed biosecurity protocols, which are currently absent in the Canary Islands^85^. From a broader perspective, this study demonstrates the potential of using eDNA techniques to detect terrestrial invasive snakes, even for highly elusive species, thus providing valuable insights that can be integrated with previous studies^27–30^. Given that a multitude of snake species are imported annually^22^ and the majority of them possess low detection probability^8,26,34,86^, the utilization of eDNA detection could hold considerable potential to enhance surveillance and early detection efforts for snakes, which are currently characterized by substantial time and resource demands^87^. Furthermore, the use of eDNA for snake detection could play a pivotal role in the long-term management of these invasive species, which currently pose a significant threat to many regions worldwide, including numerous islands^23,24^, and which have not been successfully eradicated^88,89^ or even controlled on a large scale anywhere in the world. This persistent failure underscores an urgent need to explore and adopt novel methodologies to enhance management efficacy.

## Conclusion and future work

The purpose of this research was to initiate the study of the use of eDNA techniques in the detection of *L. californiae*, an objective that aims to guide the control given the elusive behaviour of the species. In order to achieve this, we designed and tested primers for the COI subunit, allowing a short fragment of DNA from *L. californiae* to be amplified, but not from the coexisting reptiles. Given the relative paucity of development in eDNA sampling protocols for terrestrial environments in comparison to the aquatic counterparts, our comparative analysis on the probability of detecting *L. californiae* eDNA from swab samples from ACOs made of five different materials, soil underneath ACOs, random soil, and boot swab samples, suggested that swab samples under ACOs represent the most effective sampling protocol currently available (particularly effective with corrugated steel plates and corrugated bitumen sheets). Furthermore, we demonstrated that the detection rate remains consistent irrespective of the intensification of the sampling regime or the number of ACOs. However, the detection rate might be enhanced if the sampling is conducted during the months of February and March. Additionally, we concluded that, under controlled conditions, and during the seven days after an individual *L. californiae* was in direct contact with the substrate, eDNA degradation does not occur.

In order to implement the use of eDNA techniques in the routine management of *L. californiae*, further work is needed to optimise detection protocols (including refining primer specificity or eDNA amplification protocols), sampling techniques (including further research on the type of samples to be collected in the field and their location to increase detection rate), as well as to deepen the knowledge on the species eDNA accumulation and degradation in natural environments. The future development of these techniques to infer *L. californiae* density could be of great use for conservation practitioners.

## Electronic supplementary material

Below is the link to the electronic supplementary material.

Supplementary Material 1

## Data Availability

The datasets generated during and/or analysed during the current study are available at Figshare and have the following DOI number: 10.6084/m9.figshare.28202660.v1.
