# Supplementary material for: Validation of eDNA methods for managing the terrestrial invasive snake Lampropeltis californiae on the Canary Islands
Source: Sci Rep. 2025 Apr 23;15:14116. doi: 10.1038/s41598-025-96387-8 (PMC12018960; doi:10.1038/s41598-025-96387-8)
Supplement: Supplementary file 1 — Supplementary Material 1 [file 41598_2025_96387_MOESM1_ESM.docx]

VALIDATION OF eDNA METHODS FOR MANAGING THE terrestrial invasive snake LAMPROPELTIS CALIFORNIAE ON THE CANARY ISLANDS

# Supplementary INFORMATION i

Table S1. Codes, species, geographical origin, and references of the *Lampropeltis* sequences available in GenBank up to December 2023 and used for the design of specific primers for *Lampropeltis californiae* COI gene.

| **Code** | **Species** | **Origin** | **Reference** |
| --- | --- | --- | --- |
| ASCRA179-08.COI-5P | *L. triangulum* | Canadian Museum of Nature | ASCRA179-08.COI-5P |
| AY122737 | *L. getula* | Zoological museum of Zurich | Utiger et al. (2002) |
| AY122738 | *L. getula* | Zoological museum of Zurich | Utiger et al. (2002) |
| KC750812 | *L. getula* | USA | Campbell et al. not published |
| KU985672 | *L. getula* | México, South Baja California, Santiago | Chambers & Hebert (2016) |
| KU985960 | *L. getula* | México, North Baja California, Jaraguay | Chambers & Hebert (2016) |
| KU985981 | *L. getula* | México, South Baja California, San Ignacio | Chambers & Hebert (2016) |
| KU986082 | *L. getula* | California, San Diego | Chambers & Hebert (2016) |
| KU986273 | *L. getula* | México, South Baja California, Santiago | Chambers & Hebert (2016) |
| MH274238 | *L. getula* | North California, Dare | Mulcahy et al. (2022) |
| MH274239 | *L. getula* | Maryland, Charles, Nanjemoy | Mulcahy et al. (2022) |
| MH274240 | *L. triangulum* | Virginia, Fauquier | Mulcahy et al. (2022) |
| MH 274237 | *L. extenuata* | Florida, Hernando | Mulcahy et al. (2022) |

# Supplementary INFORMATION iI

## Alternative methods for soil samples processing

In order to reduce the time taken for processing soil samples in the laboratory ^4^, we took samples from the tenth hour of the first day and the seventh day of each terrarium (see main text for details of the experiment) to compare three alternative methods for processing these samples. The first method followed the protocol proposed by Matthias et al. ^4^, in which we shook the sample tube, transferred 15 mL of its content to another 50 mL Falcon tube, which we filled to 45 mL with ultrapure water (Ultra-Pure Water System, Milli-Q®, Merck, USA), and then inverted and vigorously shaken it for *c.* 30 s. We left the tube in a vertical position at 4 °C for 24 h to allow the sample to settle. Thereafter, we filtered 5 mL of the supernatant using 10-20 µm Whatman paper (VWR, Germany), which was positioned as a funnel on top of a 50 mL tube. We then cut the central section of the paper and placed it in a 1.5 mL tube. The second approach consisted in replicating the decanting process previously described, immersing a sterile swab in the supernatant, cutting its tip and dropping it in a 1.5 mL tube to extract the DNA as in the previous case. The third method involved transferring the substrate from each tube to another 2 ml tube, which we stored at -20 °C. We then rubbed a sterile cotton swab, moistened with ultrapure water (Ultra Pure Water System, Milli-Q®, Millipore®, USA), on the walls of the empty tube, and then cut and dropped its tip in a 1.5 mL tube for subsequent DNA extraction with the same protocol previously mentioned. We extracted the DNA from all cases using the commercial kit E.Z.N.A.® Tissue DNA (Omega, Bio-tek Inc., USA), following manufacturer's protocol. We amplified DNA from each protocol using conventional PCR (see main text for PCR conditions). Only the third method produced positive snake detections (Fig. S2.1).


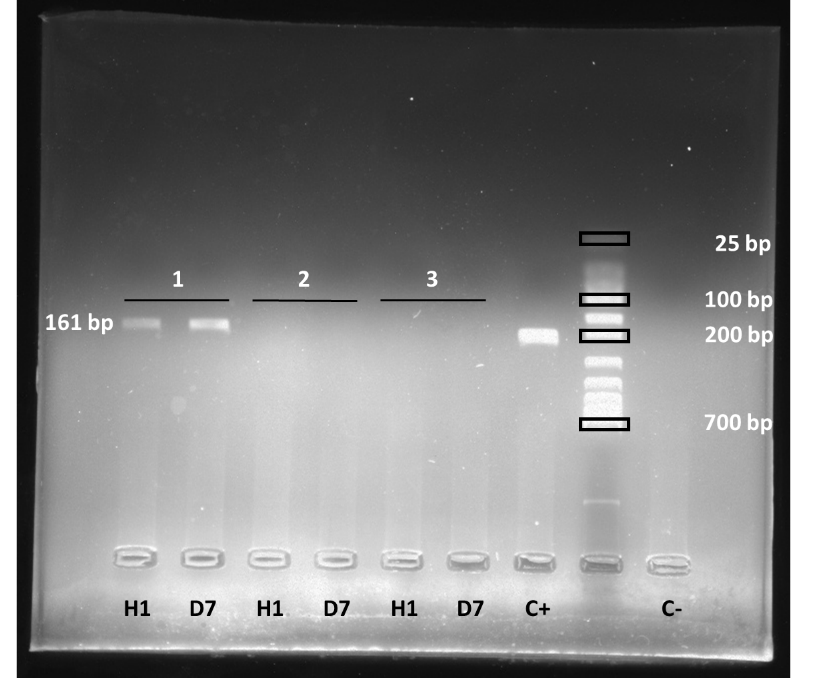


**Fig. S1.** Results of the electrophoresis performed to visualize the amplification of *Lampropeltis californiae* eDNA in terraria substrate collected in the first hour (H1) and the seventh day (D7) after putting a snake in terraria, extracted by (1) rubbing the swab on the wall of the tube, (2) soaking the swab in the supernatant, and (3) using Whatman paper after filtering (see Supplementary Material II for more details). C+ and C- refers to the positive (*L. californiae* DNA) and negative controls, respectively. We used the 25–700 bp DNA marker LADD-DN1-500 (OXGEN™).

# supplementary INFORMATION III

**Table S2.** *Lampropeltis californiae* eDNA positive samples (denoted with the number 1 in each terrarium) detected by using conventional PCR techniques from samples collected in terraria 1 to 4 (T1-T4) prior the addition of *L. californiae* individuals to terraria (0 h, negative controls), at different hours after releasing the snakes in them during the first day (from 1 to 10 h), and up to the seventh day before removing the snakes from the terraria.

| **Sample** | |  | **Terraria** | | | |
| --- | --- | --- | --- | --- | --- | --- |
| Day | Hour |  | T1 | T2 | T3 | T4 |
| 1 | 0 h |  | 0 | 0 | 0 | 0 |
|  | 1 h |  | 0 | 0 | 1 | 0 |
|  | 2 h |  | 1 | 1 | 1 | 0 |
|  | 3 h |  | 0 | 1 | 0 | 0 |
|  | 4 h |  | 0 | 1 | 0 | 0 |
|  | 5 h |  | 1 | 1 | 0 | 0 |
|  | 6 h |  | 1 | 1 | 0 | 0 |
|  | 7 h |  | 1 | 1 | 0 | 0 |
|  | 8 h |  | 0 | 1 | 0 | 1 |
|  | 9 h |  | 1 | 1 | 0 | 0 |
|  | 10 h |  | 1 | 1 | 1 | 1 |
| 2 |  |  | 0 | 1 | 1 | 1 |
| 3 |  |  | 0 | 1 | 0 | 1 |
| 4 |  |  | 1 | 1 | 1 | 0 |
| 5 |  |  | 0 | 1 | 1 | 1 |
| 6 |  |  | 0 | 1 | 0 | 0 |
| 7 |  |  | 1 | 1 | 0 | 0 |

# REFERENCES

1. Utiger, U. *et al.* Molecular systematics and phylogeny of Old and New World ratsnakes, *Elaphe* Auct., and related genera (Reptilia, Squamata, Colubridae). *Russ*. *J Herpetol* **9**, 105–124. <https://doi.org/10.30906/1026-2296-2002-9-2-105-124> (2021).

2. Chambers, E. A. & Hebert, P. D. N. Assessing DNA barcodes for species identification in North American reptiles and amphibians in natural history collections. *PLoS One* **11**, e0154363. https://doi.org/10.1371/journal.pone.0154363 (2016).3. Mulcahy, D. G. *et al.* DNA barcoding of the National Museum of Natural History reptile tissue holdings raises concerns about the use of natural history collections and the responsibilities of scientists in the molecular age. *PLoS One* **17**, e0264930. https://doi.org/10.1371/journal.pone.0264930

2022).

4. Matthias, L., Allison, M. J., Maslovat, C. Y., Hobbs, J. & Helbing, C. C. Improving ecological surveys for the detection of cryptic, fossorial snakes using eDNA on and under artificial cover objects. *Ecol Indic* **131**, 108187. [https://doi.org/10.1016/j.ecolind.2021.108187](https://doi.org/10.1016/j.ecolind.2021.108187) (2021).
